# The Evolution of the Cytochrome *c*_6_ Family of Photosynthetic Electron Transfer Proteins

**DOI:** 10.1093/gbe/evab146

**Published:** 2021-06-24

**Authors:** Barnaby Slater, Darius Kosmützky, R Ellen R Nisbet, Christopher J Howe

**Affiliations:** Department of Biochemistry, University of Cambridge, United Kingdom

**Keywords:** photosynthesis, evolutionary model, phylogeny

## Abstract

During photosynthesis, electrons are transferred between the cytochrome *b*_6_*f* complex and photosystem I. This is carried out by the protein plastocyanin in plant chloroplasts, or by either plastocyanin or cytochrome *c*_6_ in many cyanobacteria and eukaryotic algal species. There are three further cytochrome *c*_6_ homologs: cytochrome *c*_6A_ in plants and green algae, and cytochromes *c*_6B_ and *c*_6C_ in cyanobacteria. The function of these proteins is unknown. Here, we present a comprehensive analysis of the evolutionary relationship between the members of the cytochrome *c*_6_ family in photosynthetic organisms. Our phylogenetic analyses show that cytochromes *c*_6B_ and *c*_6C_ are likely to be orthologs that arose from a duplication of cytochrome *c*_6_, but that there is no evidence for separate origins for cytochromes *c*_6B_ and *c*_6C_. We therefore propose renaming cytochrome *c*_6C_ as cytochrome *c*_6B_. We show that cytochrome *c*_6A_ is likely to have arisen from cytochrome *c*_6B_ rather than by an independent duplication of cytochrome *c*_6_, and present evidence for an independent origin of a protein with some of the features of cytochrome *c*_6A_ in peridinin dinoflagellates. We conclude with a new comprehensive model of the evolution of the cytochrome *c*_6_ family which is an integral part of understanding the function of the enigmatic cytochrome *c*_6_ homologs.

## Introduction

### The Cytochrome *c*_6_ Family of Proteins

Significance

The cytochrome *c*_6_ family of proteins plays an essential role in photosynthetic electron transfer, but the evolutionary relationships among the members of the family remain unclear. We show that a previously drawn distinction between cytochromes *c*_6B_ and *c*_6C_ probably reflects taxon sampling, that cytochromes *c*_6BC_ arose from cytochrome *c*_6_, and that cytochrome *c*_6A_ subsequently arose from cytochrome *c*_6B_ after the divergence of the green photosynthetic lineage. These conclusions, together with a survey of the distribution of the family among eukaryotes, give us a much better understanding of the evolution of this important protein family.

Photosynthesis is one of the most important processes in the natural world and has played a vital role in shaping the planet and its atmosphere. One essential feature of oxygenic photosynthesis is the photosynthetic electron transfer chain (PETC), where the oxidation of water to generate reducing equivalents and chemical energy as ATP is driven through light energy absorption. In the plant PETC, electrons can be transferred between the cytochrome *b*_6_*f* complex and photosystem I by the copper-containing protein plastocyanin (Gross 1993). Many cyanobacteria and eukaryotic algae have an alternative electron transfer protein as a substitute for plastocyanin, the hemoprotein cytochrome *c*_6,_ which is used when copper is not readily available (Wood 1978). It is believed that cytochrome *c*_6_ is a more ancient protein than plastocyanin, with the latter evolving after increasing atmospheric oxygen concentrations led to a decrease in the ready availability of iron in the environment (De la Rosa et al. 2002). Green plants were believed to have lost cytochrome *c*_6_, retaining only plastocyanin (Kerfeld and Krogmann 1998).

**Fig. 1.**
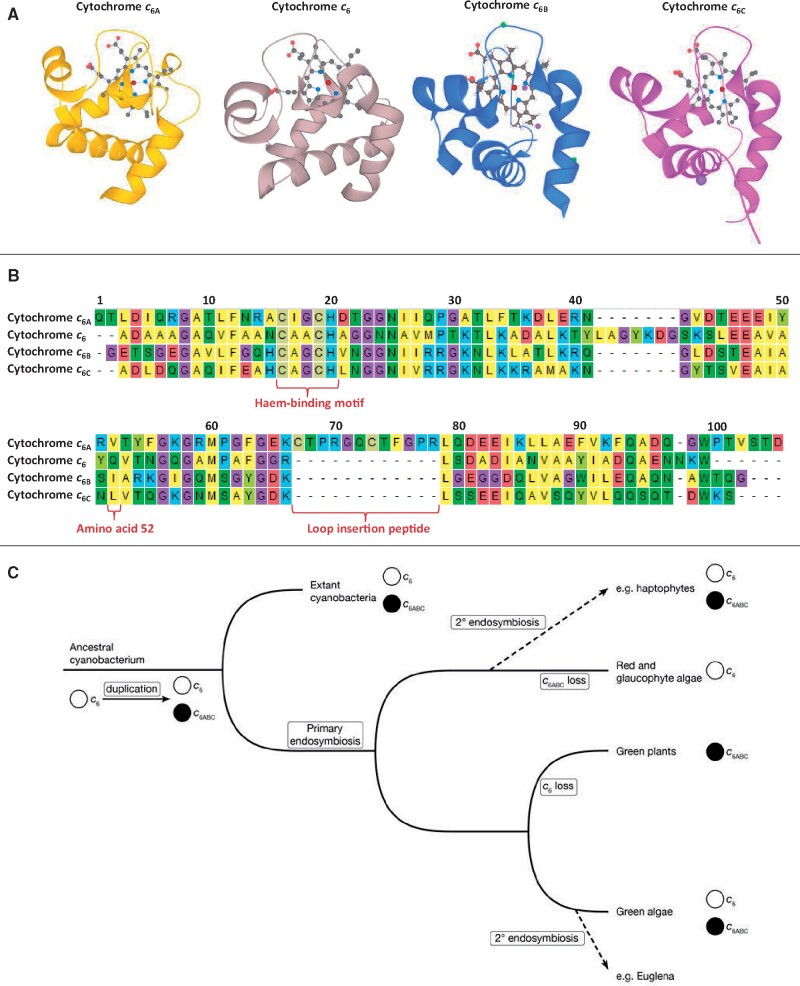
(*A*) X-ray crystal structures of cytochromes *c*_6A_ (yellow), *c*_6_ (beige), *c*_6B_ (blue), and *c*_6C_ (magenta). Secondary structure for each protein is shown in ribbon form, and the haem prosthetic groups shown in ball and stick (carbon—black, oxygen—red, nitrogen—blue, iron—deep red, and sodium—purple). (*B*) Protein sequence alignment of cytochromes *c*_6A_, *c*_6_, *c*_6B_, and *c*_6C_ from *A. thaliana* (accession Q93VA3.1), *Synechococcus* sp. PCC 7002 (accession O30881.1), *Synechococcus* WH8103 (CRY92441.1), and *Synechococcus* sp. PCC 7002 (accession AAN03578.1) respectively. The sequences have their putative signal peptides excluded. Amino acids are colored with yellow—hydrophobic residues, green—polar residues, beige—cysteines, blue—positively charged residues, and red—negatively charged residues, and the haem-binding motif (CXXCH), the LIP, and amino acid 52 are indicated below the alignment. Figure uses crystallography and sequence data from Marcaida et al. (2006), Bialek et al. (2009), and Zatwarnicki et al. (2014) (PDB cytochrome c6: 3DR0, c6A: 2CE0, c6B: 4KMG, c6C: 4EIE). (*C*) Current model of cytochrome *c*_6_ family evolution in photosynthetic organisms. Adapted from Howe et al. (2016).

However, in 2002 a homolog of cytochrome *c*_6_ was found in green plants (Gupta et al. 2002; Wastl et al. 2002). This protein was subsequently named cytochrome *c*_6A_ (Wastl et al. 2004; fig. 1*A*). The sequence of cytochrome *c*_6A_ was found to be highly similar to that of *c*_6_, with a major difference that cytochrome *c*_6A_ contains a 12-amino acid insertion in a loop region of the protein (fig. 1*B*). This insertion has been named the loop insertion peptide (LIP) (Howe et al. 2006), and contains two cysteines that form a disulfide bridge (Marcaida et al. 2006). Further homologs of cytochrome *c*_6A_ (in addition to the conventional cytochrome *c*_6_) were then discovered in cyanobacteria, and named cytochromes *c*_6B_ and *c*_6C_ (Nomura 2001; Bialek et al. 2008; fig. 1*A* and *B*). These cytochromes were split into B and C homologs based on a phylogenetic analysis, which showed that cytochrome *c*_6B_ shared a more recent common ancestry with cytochrome *c*_6A_, and cytochrome *c*_6C_ shared a more recent common ancestor with cytochrome *c*_6_ (Bialek et al. 2008).

Cytochromes *c*_6A_, *c*_6B_, and *c*_6C_ have a redox midpoint potential around 200 mV lower than cytochrome *c*_6_, suggesting that cytochromes *c*_6A_, *c*_6B_, and *c*_6C_ are unable to oxidize cytochrome *f* and have a different function from cytochrome *c*_6_ (Molina-Heredia et al. 2003; Bialek et al. 2008, 2014). This suggestion of a difference in function was supported by studies on the reaction between cytochrome *c*_6A_ and photosystem I in vitro and the demonstration that plastocyanin is essential in plants (Molina-Heredia et al. 2003; Weigel et al. 2003). The difference in redox midpoint potential between cytochromes *c*_6A_ and *c*_6_ is proposed to be largely due to a single amino acid residue, found at position 52 in *Arabidopsis thaliana* cytochrome *c*_6A_ (Marcaida et al. 2006; fig. 1*B*). In the low redox midpoint potential cytochrome *c*_6_-like proteins, this residue is hydrophobic (leucine, isoleucine, or valine), with cytochrome *c*_6_ having a conserved glutamine in the same position. Substituting the *A. thaliana* cytochrome *c*_6A_ valine 52 with a glutamine has been shown to increase the redox midpoint potential of the protein by around 100 mV (Worrall et al. 2007). The function of these low redox midpoint cytochrome *c*_6_-like proteins is currently unclear, though a role in alternative pathways in electron transfer has been proposed (Howe et al. 2016).

### The Current Model of Cytochrome *c*_6_ Family Ancestry

The current hypothesis for the evolution of the cytochrome *c_6_* family in photosynthetic organisms has been outlined by Howe et al. (2016) (fig. 1*C*). The model suggested that duplication(s) of cytochrome *c*_6_ in an ancestral cyanobacterium led to the genesis of the low redox midpoint potential cytochromes grouped under the umbrella-term cytochrome *c*_6ABC_. Phylogenetic analysis by Bialek et al. (2008) suggested that at least two duplications had occurred in cyanobacteria, resulting in cytochromes *c*_6B_ and *c*_6C._ Following primary endosymbiosis, cytochrome *c*_6_ was lost in the green plant lineage leaving only a low redox midpoint potential sequence, cytochrome *c*_6A_. Secondary endosymbiosis involving the green lineage (e.g., as seen for *Euglena*), was believed to have failed to transfer the low redox midpoint potential cytochrome *c*_6_. In contrast, cytochrome *c*_6ABC_ was believed to have been lost in the red algal and glaucophyte lineages (which contain primary plastids) sometime after the origin of the haptophytes (containing a secondary plastid; Yoon et al. 2002), which retain both cytochrome *c*_6_ and *c*_6ABC_. A recent study, however, has identified a cytochrome *c*_6BC_ homolog in the glaucophyte *Cyanophora paradoxa* (Kleiner et al. 2021).

### Aims of the Study

With the availability of more sequence data, this study expanded the search for cytochrome *c*_6_ family sequences in a wider range of photosynthetic taxa, both prokaryotic and eukaryotic. We particularly wished to identify whether cyanobacterial cytochrome *c*_6BC_ proteins were derived from cytochrome *c*_6_ or vice versa, what the evolutionary relationship is between cytochrome *c*_6B_ and cytochrome *c*_6C_, and how widely distributed the cytochrome *c*_6ABC_ family is among eukaryotes.

## Results

### Mapping Cytochromes *c*_6_, *c*_6B_, and *c*_6C_ on an Established Cyanobacterial Species Tree Shows That *c*_6B_ and *c*_6C_ Are Orthologs That Arose from a Single *c*_6_ Gene Duplication Event

**Fig. 2.**
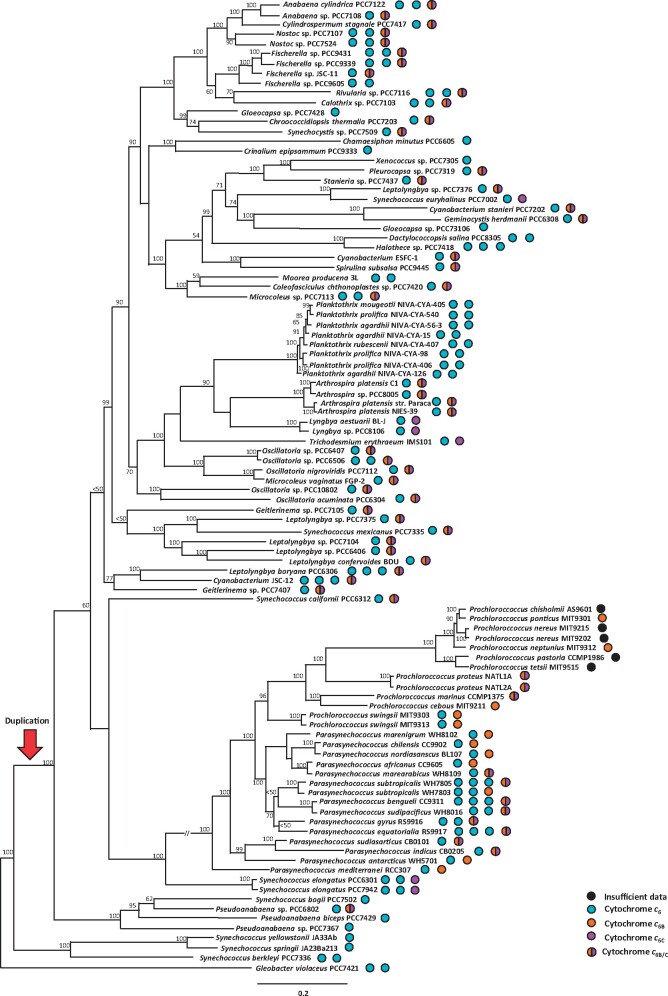
Cyanobacterial species tree with presence of cytochromes *c*_6_, *c*_6B_, and *c*_6C_ mapped onto it (colored blue, orange, and magenta, respectively). Circles with both orange and magenta semicircles contain a putative low redox midpoint potential cytochrome *c*_6_, which was not included in Bialek et al. (2008). Proteins represented by orange or purple full circles were described as *c*_6B_ or *c*_6C_, respectively by Bialek et al. (2008). Black circles represent species whose peptide or nucleotide data are not readily available to probe. Tree is reproduced from Walter et al. (2017). The potential branch where neofunctionalisation led to the evolution of cytochrome *c*_6B_ is indicated with a red arrow. Scale bar represents branch length. Bootstrap values were calculated as a percentage using 1,000 iterations Walter et al. (2017). Accessions of the sequences used for this figure can be found in supplementary table 1, Supplementary Material online.

To examine the distribution of cytochromes *c*_6_, *c*_6B_, and *c*_6C_ across cyanobacteria, the presence or absence of *c*_6_ family cytochrome sequences was mapped onto a phylogenetic tree of cyanobacterial species inferred from a concatemer of conserved sequences (Walter et al. 2017; fig. 2). This phylogenetic framework represents a comprehensive phylogenomic analysis of cyanobacteria based on 31 conserved gene markers, using Maximum Likelihood (ML). Putative cytochrome *c*_6B/C_ sequences were found by database searching and defined as cytochrome *c*_6B/C_ if they had both an appropriately located haem-binding motif (CXXCH; Barker and Ferguson 1999) and a valine, leucine, or isoleucine rather than glutamine at the equivalent of position 52 in *A. thaliana*. (The presence of valine at this position was linked to a lower redox midpoint potential relative to cytochrome *c*_6_ [Worrall et al. 2007; Bialek et al. 2008].)

With this expanded sampling, the sequences identified as cytochrome *c*_6B_ by Bialek et al. (2008) were found exclusively in one clade, which contained cyanobacteria of the genera *Prochlorococcus* or *Parasynechococcus*, as shown in figure 2. In contrast, the sequences previously identified as cytochrome *c*_6C_ (Bialek et al. 2008) were widespread across the cyanobacterial species tree, but not found in the genera *Prochlorococcus* or *Parasynechococcus*. In addition, no species in the tree contained more than one cytochrome *c*_6B/C_-like sequence. These observations suggest that the prior separation observed between cytochrome *c*_6B_ and *c*_6C_ could be accounted for by taxon sampling, without needing to propose them as two separate families.

The cyanobacterial tree shows a split (labeled with a red arrow) separating taxa which have only cytochrome *c*_6_ from those which also have a cytochrome *c*_6B_ or *c*_6C_ sequence. This branch point separates the basally diverging *Gloeobacter violaceus* PCC7421 (Criscuolo and Gribaldo 2011; Mareš et al. 2013) and a few other taxa from the rest. This distribution suggests that cytochrome *c*_6_ appeared first, and that cytochromes *c*_6B_ and *c*_6C_ may have arisen through duplication and neofunctionalization of cytochrome *c*_6_.

### A Phylogenetic Tree Using a Wider Taxon Selection Suggests a Single Origin for Cytochromes *c*_6B_ and *c*_6C_

**Fig. 3.**
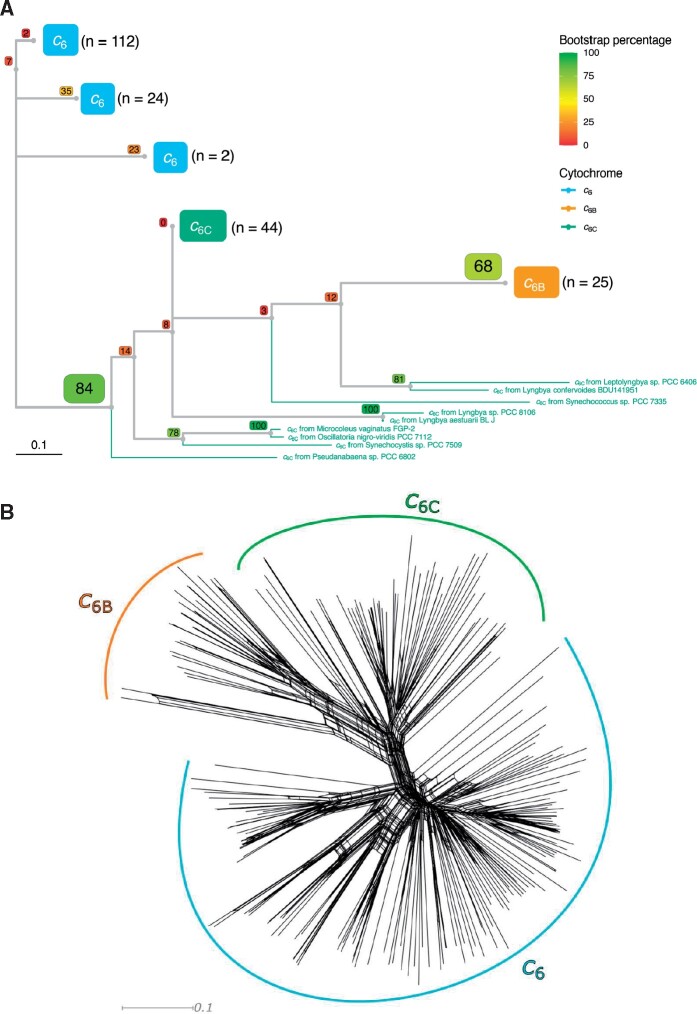
Condensed phylogenetic tree (*A*) and Neighbor-Net splits graph (*B*) inferred from an alignment of cytochrome *c*_6_, *c*_6B_, and *c*_6C_ peptide sequences from cyanobacterial species (colored blue, orange, and green, respectively). Alignments were performed using MUSCLE algorithm and can be found in the supplementary information along with accessions for each sequence used. The phylogenetic tree was built using ML inference using a WAG model with Gamma distribution and invariable sites (WAG + G + I). Bootstrap values for each branch point, using 100 iterations, are shown in colored boxes. The *n* value next to each group represents the number of sequences found within each clade. The full tree is shown in supplementary figure 1, Supplementary Material online and the alignment from which the tree was inferred can be found in supplementary table 2, Supplementary Material online. SplitsTree4 was used to obtain the Neighbor-Net splits graph.

To investigate further the hypothesis of cytochromes *c*_6B_ and *c*_6C_ being duplicates of cytochrome *c*_6_ with a new function, a phylogenetic tree was inferred from an alignment of cytochromes *c*_6_, *c*_6B_, and *c*_6C_ sequences covering all the organisms used in two independent phylogenetic analyses of the cyanobacterial lineage (Schirrmeister et al. 2015; Walter et al. 2017). A condensed tree is shown in figure 3*A* (the full tree can be found in supplementary fig. 1, Supplementary Material online). As the sequences are short, a Neighbor-Net analysis was also performed (fig. 3*B*) and showed that the data were treelike, and that tree-based phylogenetic analysis was appropriate (Huson and Bryant 2006).

The cytochromes predicted to have a low redox midpoint potential, including those assigned as cytochromes *c*_6B_ and *c*_6C_ previously, all grouped to the exclusion of the predicted cytochrome *c*_6_ sequences in both the phylogenetic tree (bootstrap value of 84%) and the Neighbor-Net analysis, and maintained a similar general topology to that of the cyanobacterial tree of figure 2. This distribution showed cytochrome *c*_6B_ as a clade derived from within the cytochrome *c*_6C_ clade, as with figure 2. Once again, there was no evidence of both cytochromes *c*_6B_ and *c*_6C_ within the same organism. (*Crocosphaera watsonii* has been shown to have two low redox midpoint potential cytochrome *c*_6_ sequences, but both were assigned as cytochrome *c*_6C_ in prior studies [Bialek et al. 2008].) These observations suggest a single origin for the cytochrome *c*_6BC_ family, and that they are orthologs rather than paralogs. It is worth noting that the bootstrap values in this tree were considerably lower than those in the tree inferred by Walter et al. (2017), which is to be expected with a larger number of taxa for sequences of short sequence length (Rokas and Carroll 2005) such as with the cytochrome *c*_6_ family peptides. However, the tree-like appearance of the Neighbor-Net analysis inferred from the same sequence alignment suggests that phylogenetic inference is appropriate.

The neighboring open-reading frames of cytochromes *c*_6B_ and *c*_6C_ in cyanobacterial genomes were compared (data not shown). Most of the species that possess a cytochrome *c*_6B_ were observed to have neighboring genes coding for Nif1 domain-containing, YciI family, or DUF3136 domain-containing proteins, except for *Prochlorococcus marinus* str. MIT 9312 and MIT 9301. The most closely related species possessing a cytochrome *c*_6C_ had different neighboring genes from those in the cytochrome *c*_6B_ species. However, it is difficult to determine whether this difference in genetic neighborhoods is due to cytochromes *c*_6B_ and *c*_6C_ being paralogs, or due to the overall similarity between the genomes of species containing cytochrome *c*_6B_, which comprise only two genera of cyanobacteria. Additionally, there was a high diversity of genetic neighborhoods amongst cytochrome *c*_6C_ possessing species, which represent a wider range of cyanobacteria. There was therefore no evidence from synteny to indicate that cytochromes *c*_6B_ and *c*_6C_ are paralogs.

Taken together, there is no evidence that would support a functional differentiation between cytochromes *c*_6B_ and *c*_6C_. Cytochrome *c*_6B_ is found only in a clade of organisms known for a high protein substitution rate (Dufresne et al. 2005), cytochromes *c*_6B_ and *c*_6C_ both have a low redox midpoint potential, share common ancestry to the exclusion of cytochrome *c*_6_, and are not found together in one organism. The distinction between cytochromes *c*_6B_ and *c*_6C_ does not seem to represent functional divergence, and we propose to refer to all as cytochromes *c*_6B_ in future.

### Distribution of Cytochrome *c*_6_ Family Members across Photosynthetic Eukaryotes

The recent sequencing of genomes and transcriptomes of a wider range of eukaryotic photosynthetic organisms allowed for a more thorough search for *c*_6_-like cytochromes, including cytochrome *c*_6A_. Protein and nucleotide databases across eukaryotes were searched using BLAST for cytochrome *c*_6_ family sequences. Sequences recovered were defined as cytochrome *c*_6A_ if they contained a hydrophobic residue (valine, leucine, or isoleucine) at the equivalent of position 52, indicating a low redox midpoint potential, and an insertion containing a disulfide bridge (the LIP) in the loop region compared with cytochrome *c*_6_. Sequences recovered were defined as cytochrome *c*_6B_ if they contained the hydrophobic residue implying a low redox midpoint but not the LIP.

#### Distribution of Cytochrome c6 after Primary Endosymbiosis

**Fig. 4.**
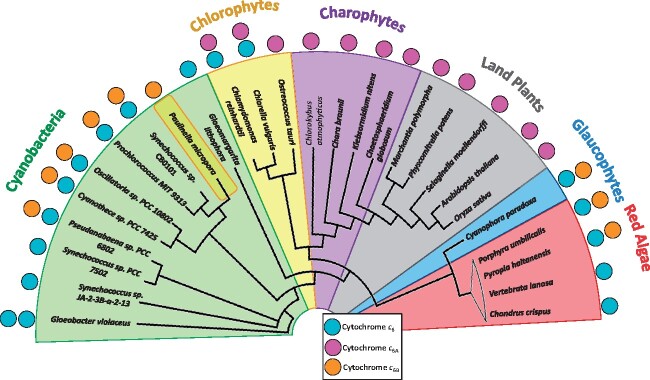
Distribution of *c*_6_-type cytochromes across photosynthetic lineages. Presence of a colored circle adjacent to a species name indicates that a sequence of the relevant cytochrome was found in sequence database searches, with multiple copies of the same colored circle indicating a potential paralog. *Paulinella chromatophora* is highlighted to segregate it from the cyanobacteria. Phylogenetic tree branch lengths are not to scale. Diagram based on phylogenetic tree from Ponce-Toledo et al. (2017). Accession numbers for individual gene sequences can be found in supplementary table 3, Supplementary Material online.

The distribution of cytochromes *c*_6_, *c*_6B_, and *c*_6A_ across cyanobacteria and in eukaryotes after primary endosymbiosis was mapped onto a phylogenetic tree based on an alignment of concatemers of plastid genes and cyanobacterial homologs (fig. 4). The presence of cytochromes *c*_6_ and *c*_6B_ in the glaucophyte and red algal lineages suggests that the cyanobacterium involved in the primary endosymbiosis event contained both a cytochrome *c*_6_ and a *c*_6B_.

The results indicate that after primary endosymbiosis, cytochrome *c*_6B_ was replaced by cytochrome *c*_6A_ in the green plant and algal lineage. This suggests that cytochrome *c*_6A_ was derived from cytochrome *c*_6B_, possibly through an insertion of the LIP early in the green chloroplast lineage. The insertion of the LIP into an existing sequence rather than duplication and divergence is supported by the observation that no species have been found to contain both a cytochrome *c*_6A_ and a *c*_6B_ sequence. Although cytochrome *c*_6_ was identified in some chlorophyte species, it was not identified in any charophytes or land plants, suggesting that the loss of cytochrome *c*_6_ occurred in the ancestor to the charophyte lineage. This in turn has resulted in land plants exclusively containing cytochrome *c*_6A_. In contrast, the glaucophytes and many red algal species have retained the original cytochrome *c*_6_. However, some red algal species (though not all) appeared to have lost cytochrome *c*_6B_, for example *Chondrus crispus*. Finally, the eukaryotic protist *Paulinella* contains a cytochrome *c*_6B_-like sequence. This is likely to reflect the recent, independent primary endosymbiosis of a cyanobacterium that gave rise to the *Paulinella* chloroplast (Marin et al. 2005; Yoon et al. 2006), although the *Paulinella* line also appears to have lost cytochrome *c*_6_.

#### Distribution of Cytochrome c6 Family Members after Secondary Endosymbioses

Many photosynthetic eukaryotes contain chloroplasts of secondary origin. We therefore searched for the presence of cytochromes *c*_6_, *c*_6B_, and *c*_6A_ in these organisms. *Euglena gracilis*, which contains a chloroplast of secondary green origin (Turmel et al. 2009), was predicted to contain a cytochrome *c*_6A_ sequence in addition to cytochrome *c*_6_ (Novák Vanclová et al. 2020). The chlorarachniophytes, a class of Rhizaria with a secondary chloroplast of green origin (Rogers et al. 2007), on the other hand only had a cytochrome *c*_6_ sequence and no evidence of cytochrome *c*_6A_. This suggests that either the green algal endosymbiont of the chlorarachniophytes did not have cytochrome *c*_6A_ or that the gene was lost after secondary endosymbiosis.

Different organisms with a secondary red chloroplast also varied in cytochrome *c*_6_ family gene distribution. Haptophytes, cryptomonads, and some ochrophytes, which contain a chloroplast of a red algal origin (Yoon et al. 2002), contained cytochrome *c*_6_ and *c*_6B_ sequences, as expected. However, many ochrophytes and some haptophytes and cryptomonads had no evidence of *c*_6B_ sequences. This suggests that the red algal endosymbiont to haptophytes, cryptomonads, and ochrophytes had retained cytochrome *c*_6B_, and that the gene was lost afterwards downstream in certain lineages, although widespread lateral transfer cannot be excluded.

The situation in dinoflagellate algae is complex. The peridinin dinoflagellates contain a chloroplast of secondary red origin (Dorrell and Howe 2015). Two peridinin dinoflagellates (*Amphidinium carterae* and *Symbiodinium microadriaticum*) contain a cytochrome *c*_6_ sequence and what appeared to be a cytochrome *c*_6A_ sequence. In contrast, *Karlodinium veneficum*, a fucoxanthin dinoflagellate (which obtained its chloroplast via loss of the red algal chloroplast and serial endosymbiosis of a haptophyte [Dorrell and Howe 2015; Klinger et al. 2018]), contains a cytochrome *c*_6_ and cytochrome *c*_6B_. The existence of a cytochrome *c*_6B_ in the *Karlodinium* lineage, whose chloroplast is of red algal origin, is not surprising. However, the peridinin dinoflagellate chloroplast is also of red algal origin, so the apparent existence of cytochrome *c*_6A_ in this lineage is unexpected.

**Fig. 5.**
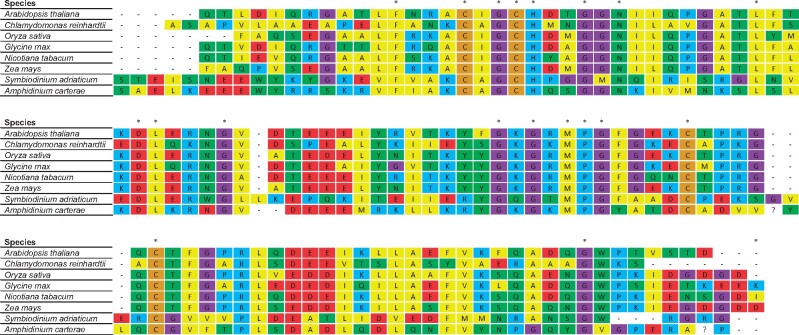
MUSCLE alignment of cytochrome *c*_6A_ from green eukaryotic lineages and the putative cytochrome *c*_6A_ sequences from the dinoflagellates *S. microadriaticum* and *A. carterae*. Amino acids are colored such that yellow—hydrophobic residues, green—polar residues, blue—positively charged residues, beige—cysteines, purple—glycines, light green—tyrosine, and red—negatively charged residues. Dashes indicate inserted gaps. Asterisks represent conserved residues. The sequences of the mature dinoflagellate proteins have been cut for a clearer depiction of the alignment, with the *S. microadriaticum* sequence beginning at amino acid 50 and the *A. carterae* sequence beginning at amino acid 123. Accessions used: *A. thaliana* (AT5G45040.1), *C. reinhardtii* (XP_001692119.1), *O. sativa* (EAZ04378.1), *G. max* (KRH50430.1), *N. tabacum* (XP_016489567.1), *Z. mays* (ACN28933.1), *S. microadriaticum* (OLP91854.1), and *A. carterae* (CF065358.1).

The sequences resembling cytochrome *c*_6A_ in peridinin dinoflagellate algae were compared with those of other cytochrome *c*_6A_ proteins (fig. 5). This revealed that the dinoflagellate sequences show some sequence similarity with the cytochromes *c*_6A_ from the green chloroplast lineage. The LIP insertion, however, shows very little sequence similarity between dinoflagellates and green plants, except for the two characteristic cysteine residues. It should also be noted that the dinoflagellate sequences are longer than the cytochrome *c*_6A_ sequences from green plants, with the first position of each of the dinoflagellate sequences in figure 5 being residues 123 and 50 for *A. carterae* and *S. microadriaticum*, respectively. These observations suggest that the putative dinoflagellate cytochrome *c*_6A_ sequences have a functional similarity to cytochrome *c*_6A_ from green plants, but are likely to have an independent origin.

A summary of the photosynthetic eukaryotes and the presence of each cytochrome *c*_6_ family sequence discovered can be found in table 1. Taxon IDs of clades searched are in supplementary table 4, Supplementary Material online, and accession numbers of the sequences found are in supplementary table 1, Supplementary Material online.

**Table 1:** The Presence or Absence of Cytochrome *c*_6_ Family Members across Photosynthetic Eukaryotes

Supergroup	Clade^a^	Plastid	Cytochrome		
			*c_6_* ^b^	*c*_6A_^b^	*c*_6B_^b^
Glaucophyta	Glaucophyta (*n* = 1)	Primary	✓ (1)	O	✓ (1)
Rhodophyta	Rhodophyta (*n* = 190)	Primary	✓ (190)	O	✓ (4)
Viridiplantae	Chlorophyta (*n* = 42)	Primary	✓ (32)	✓ (34)	O
Viridiplantae	Streptophyta (*n* = 217)	Primary	O	✓ (215)	O
SAR—Rhizaria	*Paulinella* (*n* = 3)	Primary cyanobacteria	O	O	✓ (3)
	Chlorarachniophyta (*n* = 2)	Secondary green	✓ (2)	O	O
Cryptomonads	Cryptomonads (*n* = 7)	Secondary (rhodophyta)	✓ (2)	O	✓ (1)
Haptista	Haptophyta (*n* = 6)	Secondary (rhodophyta)	✓ (6)	O	✓ (3)
SAR—Heterokonta	Ochrophyta (*n* = 80)	Secondary (rhodophyta)	✓ (78)	O	✓ (2)
SAR—Alveolata	Dinoflagellata (*n* = 7)	Secondary (rhodophyta)/Tertiary (haptophyta)	✓ (6)	✓ (2)	✓ (1)
Discoba—Euglenozoa	Euglenids (*n* = 2)	Secondary (chlorophyta)	✓ (2)	✓ (1)	O

a Where *n* represents the number of organisms searched.

b The number of sequences found is given in brackets.

### Cytochrome *c*_6A_ Arose from Cytochrome *c*_6B_ Rather Than Directly from Cytochrome *c*_6_

**Fig. 6.**
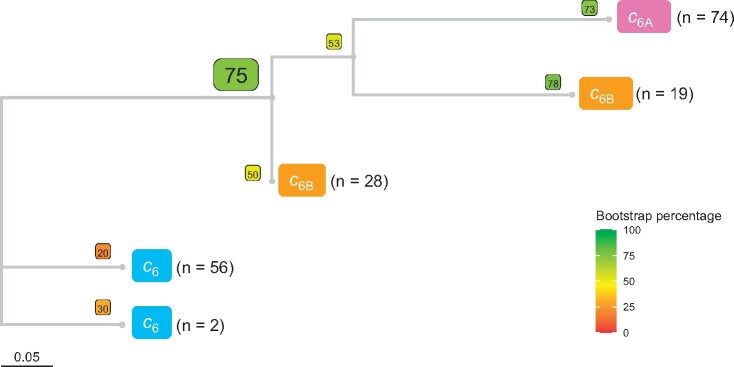
Condensed phylogenetic tree inferred from an alignment of cytochrome *c*_6_, *c*_6A_, and *c*_6B_ peptide sequences (colored blue, pink, and orange, respectively) from eukaryotic algae, green plants, and cyanobacteria. Alignments were performed using MUSCLE algorithm and can be found in the supplementary information along with accessions for each sequence used. The tree was built using ML inference using a WAG model with Gamma distribution and invariable sites (WAG + G + I). Bootstrap values for each branch point, using 100 iterations, are shown in colored boxes. The full tree is shown in supplementary figure 2, Supplementary Material online and the alignment from which the tree was inferred can be found in supplementary table 5, Supplementary Material online.

To test if cytochrome *c*_6A_ (non-dinoflagellate) arose from cytochrome *c*_6B_, rather than by independent modification of a cytochrome *c*_6_, a phylogenetic tree was inferred using cytochrome *c*_6A_ sequences from eukaryotic algae and green plants, together with cytochromes *c*_6_ and *c*_6B_ from a wide range of cyanobacteria (fig. 6 shows a condensed version of this tree, and the full tree can be found in supplementary fig. 2, Supplementary Material online). Cytochromes *c*_6A_ and *c*_6B_ grouped together to the exclusion of cytochrome *c*_6_ (bootstrap value of 75), suggesting that cytochrome *c*_6A_ shares most recent common ancestry with cytochrome *c*_6B_. This supports the conclusion above (fig. 4) that cytochrome *c*_6A_ was derived from cytochrome *c*_6B_ through an insertion event in the loop region, rather than independently of cytochrome *c*_6B_. Once again, the bootstrap values in the tree were considerably lower than those of the species tree established by Walter et al. (2017), but this is to be expected as the *c*_6_ family cytochrome sequences are short. (A Neighbor-Net splits graph for this alignment was also constructed (not shown) but was less clearly resolved and did not give any additional information.)

## Discussion

### Cytochromes *c*_6B_ and *c*_6C_ Are Orthologs

The original differentiation of cytochromes *c*_6B_ and *c*_6C_ was based on the sequence data available at the time (Bialek et al. 2008). However, now that more genomic sequence data are available, cytochrome *c*_6_ family sequences from a larger range of taxa can be analyzed. Our analysis indicates that the distinction between cytochromes *c*_6B_ and *c*_6C_ can be accounted for by taxon sampling rather than differences in function. (Although cytochromes *c*_6B_ and *c*_6C_ lie on opposite sides of the root of the cytochrome *c*_6_ family in the tree of Bialek *et al.* [2008], the placing of the root should be viewed with caution given that it depends on other *c*-type cytochromes of very different function from the cytochrome *c*_6_ family.) In addition, as the crystal structures, surface charge distribution, and redox midpoint potentials of cytochromes *c*_6B_ and *c*_6C_ are notably similar (Zatwarnicki et al. 2014; fig. 1*A* and *B*), it seems likely that cytochromes *c*_6B_ and *c*_6C_ perform a similar function and are thus orthologs.

### Two Independent Origins of *c*_6A_

Although the presence of cytochrome *c*_6A_ in plants and green algae has been known for time, the presence of cytochrome *c*_6A_ in peridinin dinoflagellates was unexpected. Dinoflagellates contain chloroplasts of secondary or tertiary origin, depending on species. The chloroplast found in *S.microadriaticum* and *A.carterae* contains peridinin, and is believed to represent the ancestral dinoflagellate chloroplast. This chloroplast was most likely obtained through secondary endosymbiosis of red algae (Dorrell and Howe 2015). Therefore, these species might be expected to contain a cytochrome *c*_6B_. Instead, the peridinin dinoflagellates contain a cytochrome *c*_6A_-like sequence. Two hypotheses for this are 1) the result of lateral gene transfer from an organism with cytochrome *c*_6A_ and the loss of the cytochrome *c*_6B_ or 2) the insertion of a LIP-like sequence into an existing cytochrome *c*_6B_ sequence. Although lateral gene transfer to dinoflagellates from other organisms has been well documented (Takishita et al. 2003; Hackett et al. 2005; Chan et al. 2012; Wisecaver et al. 2013), and it is difficult to exclude conclusively lateral transfer of cytochrome *c*_6A_ into the dinoflagellates, the low sequence similarity between the dinoflagellate *c*_6A_ and those from the green plant lineage would suggest an independent LIP insertion into cytochrome *c*_6B_ in dinoflagellates is more likely.

### The Current Model of Cytochrome *c*_6_ Family Ancestry

**Fig. 7.**
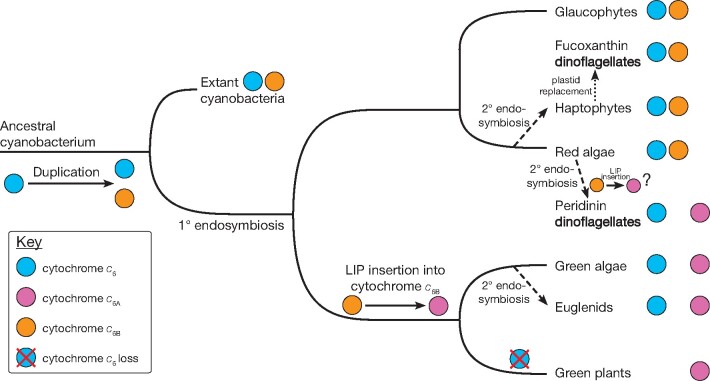
Updated ancestry model for the cytochrome *c*_6_ family in photosynthetic organisms.

The analysis of the cytochromes *c*_6_, *c*_6A_, and *c*_6B_ in this study provides a revised evolutionary model for cytochrome *c* homologs consistent with more extensive taxon sampling (fig. 7). As more anciently diverged cyanobacterial species such as *Gloeobacter* appear to contain cytochrome *c*_6_ exclusively, this suggests that the low redox midpoint potential cytochromes are more recent than cytochrome *c*_6_. A duplication of cytochrome *c*_6_, followed by point mutations that lowered the redox midpoint potential, led to the evolution of cytochrome *c*_6B_. This is supported by the presence of both cytochromes *c*_6_ and *c*_6B_ in most extant cyanobacteria today. At primary endosymbiosis, giving rise to the red, green, and glaucophyte chloroplasts, the genes were transferred to photosynthetic eukaryotes.

Red algal lineages and the glaucophytes contain cytochromes *c*_6_ and *c*_6B,_ although some red algal species have lost cytochrome *c*_6B_. In the green chloroplast lineages, cytochrome *c*_6B_ was replaced by cytochrome *c*_6A_. This was probably due to an insertion of the LIP into cytochrome *c*_6B_, as cytochrome *c*_6A_ is monophyletic within cytochrome *c*_6B_ (fig. 6). In many chlorophyte species, both cytochromes *c*_6_ and *c*_6A_ are present. In the charophytes, ancestors to the green land plants, cytochrome *c*_6_ was lost. In consequence, land plants contain only a cytochrome *c*_6A_.

Organisms containing chloroplasts of secondary origin appear have inherited their cytochrome *c*_6_ family genes from the relevant endosymbiont. The haptophytes obtained both cytochromes *c*_6_ and *c*_6B_ from the red algal chloroplast, and these genes were transferred to the fucoxanthin dinoflagellates following serial endosymbiosis. In contrast, the peridinin dinoflagellates, containing chloroplasts of secondary red origin, probably converted the cytochrome *c*_6B_ into a cytochrome *c*_6A_-like protein through the insertion of a novel LIP. With green plastid secondary endosymbiosis, genes for both cytochromes *c*_6_ and *c*_6A_ were passed to the euglenids (Novák Vanclová et al. 2020).

Overall, it is clear that the low potential cytochrome *c*_6AB_ family is widely, but not universally, present among oxygenic photosynthetic organisms. It is unlikely to be essential under all conditions, but there is no obvious environmental feature common to those organisms that retain a member of the family. The function of the cytochrome *c*_6AB_ family remains to be determined.

## Materials and Methods

### Construction of Phylogenetic Trees

A cytochrome *c*_6B_ sequence (BAD79758.1) was used for searching the “non-redundant protein sequences (*nr*)” database with the NCBI BLASTp algorithm, limiting the results to the organisms used in two independent phylogenetic analyses of the cyanobacterial lineage (Schirrmeister et al. 2015; Walter et al. 2017). A cytochrome *c*_6A_ protein sequence (AED95193.1) was used to search the *nr* protein database and the “nucleotide collection (*nr*/*nt*)” nucleic acid database with the BLASTp and tBLASTn algorithms respectively, limiting the results to the orders used in a phylogenetic analysis of green plants (Ruhfel et al. 2014). The resulting sequences of both searches were downloaded from the NCBI BLAST result page.

The retrieved peptide sequences were imported into MEGA7 (Kumar et al. 2016). Sequences were deleted from the selection if they were too short or too long to be a valid cytochrome *c*_6(A/B)_ sequence (less than 80 and more than 200 amino acids before N-terminal targeting peptide trimming) or did not have a CxxCH haem binding motif. The sequences were aligned using the MUSCLE algorithm with UPGMA clustering method and a gap-opening penalty of −2.9 and no gap extension penalty. Subsequently the putative signal peptides were trimmed from the sequences.

The WAG model with gamma distribution and invariable sites (WAG+G + I) (Whelan and Goldman 2001) was determined to be optimal for tree inference with maximum likelihood (ML) by the “Find Best DNA/Protein Models (ML)” tool in MEGA7 and was thus used in the algorithm parameters for tree inference. Statistical testing was performed using the bootstrap method with 100 iterations. The final trees were visualized using Treeio in RStudio (Wang et al. 2020). The accessions of the sequences used for inference of phylogenetic trees can be found in supplementary tables 2 and 5, Supplementary Material online.

The same aligned sequences were imported into SplitsTree4 (Huson and Bryant 2006), and the software was used to build a Neighbor-Net splits graph.

### Database Queries for Peptide Sequences

Searches for protein sequences homologous to the cytochrome *c*_6_ family, or nucleotide sequences encoding them, were performed using NCBI BLAST both in BLASTp and tBLASTn (https://www.ncbi.nlm.nih.gov/). The cytochrome *c*_6_*c*_6A_, *c*_6B_, and *c*_6C_ peptide sequences (without targeting) used in both BLASTp and tBLASTn searches were from accessions ALJ67080.1, AED95193.1, AAP99622.1, and ACB00369.1, respectively. For BLASTp searches, the database searched was “Non-redundant protein sequences (nr)” with default parameters. For tBLASTn searches, the databases searched were “nucleotide collection (nr/nt),” “Whole-genome shotgun contigs (wgs),” and “Expressed sequence tags (est)” with default parameters. Having identified a putative cytochrome *c*_6_ sequence, each organism that provided a query sequence was searched again to confirm the query as the best hit.

## Supplementary Material

Supplementary data are available at *Genome Biology and Evolution* online.

## Supplementary Material

evab146_Supplementary_Data
