# Supplementary material for: The Evolution of the Cytochrome c6 Family of Photosynthetic Electron Transfer Proteins
Source: Genome Biol Evol. 2021 Jun 24;13(8):evab146. doi: 10.1093/gbe/evab146 (PMC8358224; doi:10.1093/gbe/evab146)
Supplement: evab146_Supplementary_Data [file evab146_supplementary_data.zip › suppl figure 2 legend.pdf]

Supplementary figure 2: Phylogenetic tree inferred from an alignment of cytochrome c_6, c_{6A}, and c_{6B} peptide sequences from eukaryotic algae, higher plants and cyanobacteria (coloured blue, pink and orange respectively). Alignments were performed using Muscle algorithm. The tree was built using maximum likelihood inference using a WAG model with Gamma distribution and invariant sites (WAG+G+I). Bootstrap values for each branch point, using 100 iterations, are shown in coloured boxes. The alignment from which the tree was inferred can be found in supplementary table 5 and the condensed version of this tree is shown in figure 7.
