# Supplementary figures and images for: The Evolution of the Cytochrome c6 Family of Photosynthetic Electron Transfer Proteins
Source: Genome Biol Evol. 2021 Jun 24;13(8):evab146. doi: 10.1093/gbe/evab146 (PMC8358224; doi:10.1093/gbe/evab146)

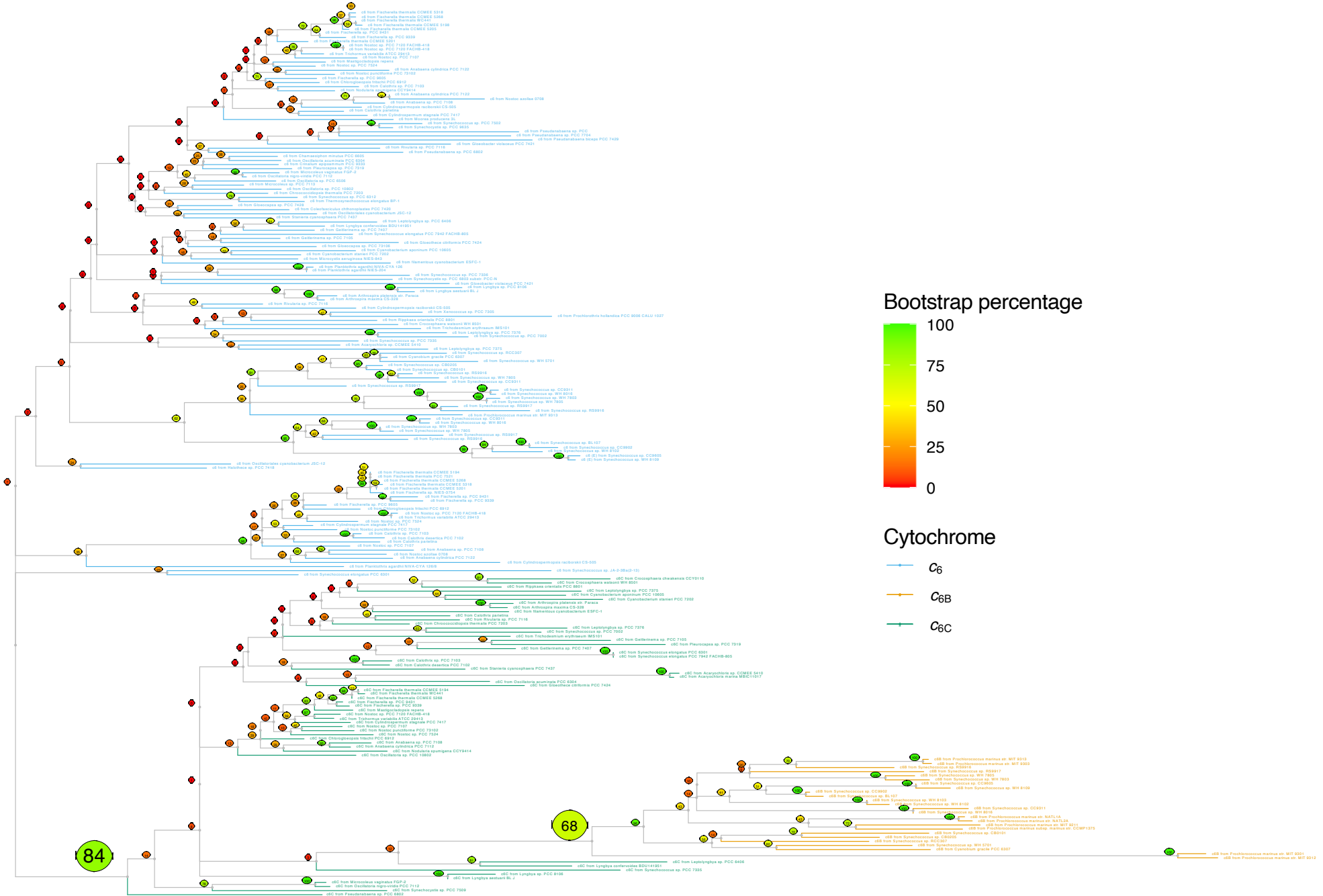

Supplement: evab146_Supplementary_Data [file evab146_supplementary_data.zip › suppl figure 1.pdf]

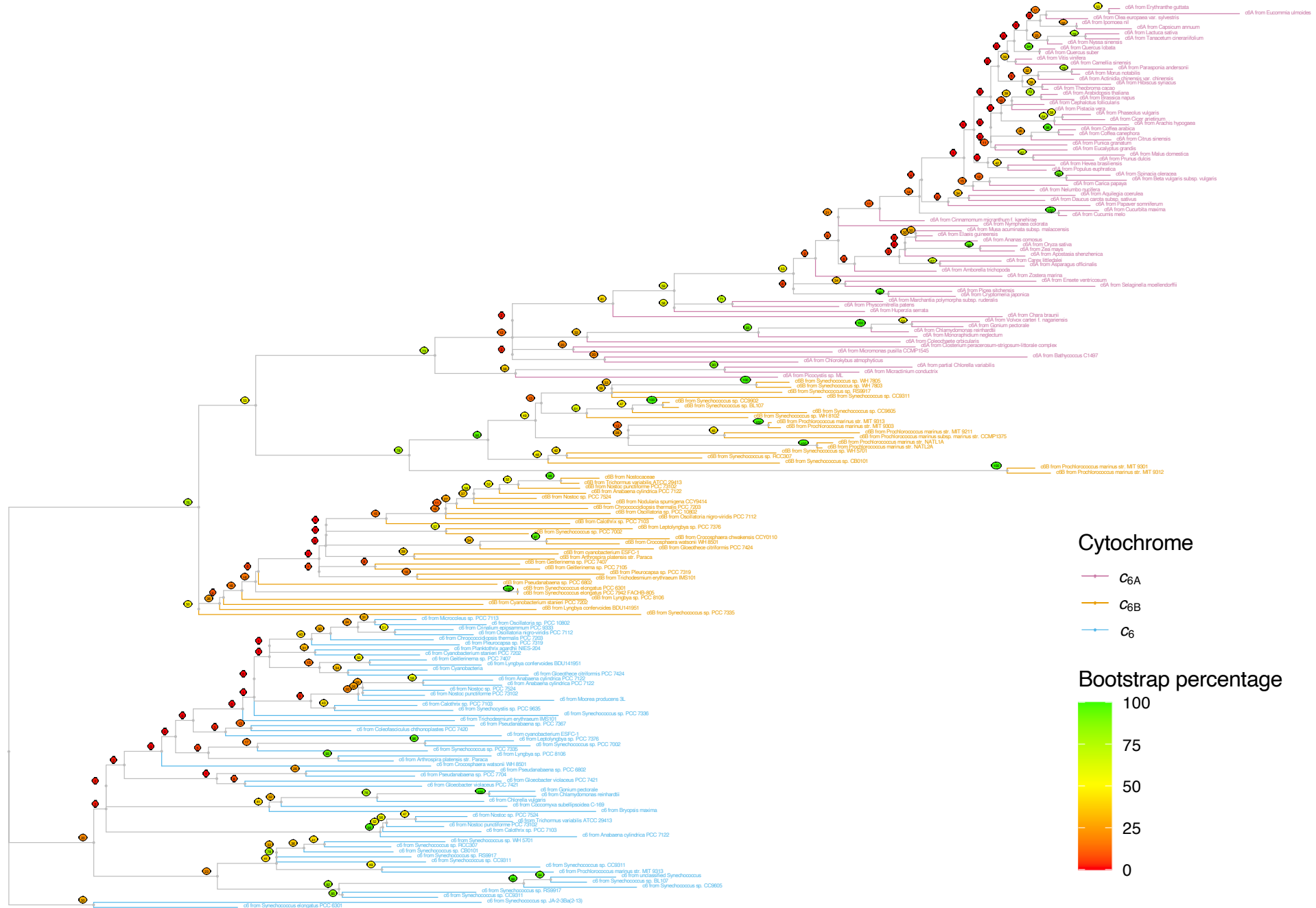

Supplement: evab146_Supplementary_Data [file evab146_supplementary_data.zip › suppl figure 2.pdf]
